# The PPAR Gamma Agonist Troglitazone Regulates Erk 1/2 Phosphorylation via a PPAR*γ*-Independent, MEK-Dependent Pathway in Human Prostate Cancer Cells

**DOI:** 10.1155/2012/929052

**Published:** 2012-02-20

**Authors:** Adrienne Bolden, Lynikka Bernard, Danielle Jones, Tunde Akinyeke, LaMonica V. Stewart

**Affiliations:** ^1^Department of Biochemistry and Cancer Biology, Meharry Medical College, Nashville, TN 37208, USA; ^2^Basic Science and Craniofacial Biology, New York University College of Dentistry, 345 East 24th Street, New York City, NY 10010, USA

## Abstract

Thiazolidinediones (TZDs) dramatically reduce the growth of human prostate cancer cells *in vitro* and *in vivo*. To determine whether the antitumor effects of TZDs were due in part to changes in the MEK/Erk signaling pathway, we examined the regulation of Erk phosphorylation by the TZD troglitazone within the PC-3 and C4-2 human prostate cancer cell lines. Western blot analysis revealed troglitazone-induced phosphorylation of Erk in both PC-3 and C4-2 cells. Troglitazone-induced increases in Erk phosphorylation were suppressed by the MEK inhibitor U0126 but not by the PPAR*γ* antagonist GW9662. Pretreatment with U0126 did not alter the ability of troglitazone to regulate expression of two proteins that control cell cycle, p21, and c-Myc. Troglitazone was also still effective at reducing PC-3 proliferation in the presence of U0126. Therefore, our data suggest that troglitazone-induced Erk phosphorylation does not significantly contribute to the antiproliferative effect of troglitazone.

## 1. Introduction

The thiazolidinediones (TZDs) are a group of high-affinity agonists for the peroxisome proliferator activated receptor gamma (PPAR*γ*) that includes the compounds ciglitazone, troglitazone (Rezulin), rosiglitazone (Avandia), and pioglitazone (Actos) [1, 2]. TZDs were initially recognized for their ability to induce adipocyte differentiation in mouse cell lines [2] and in human patients increase insulin sensitivity and reduce plasma glucose levels [3]. However, subsequent studies have shown that TZDs also reduce growth of multiple types of cancers. Micromolar concentrations of TZDs inhibit growth of tumor cells derived from the breast [4–7], bladder [8, 9], stomach [10], and colon [11–13]. Furthermore, data from our laboratory and others have shown that TZDs inhibit growth of human prostate cancer cells *in vitro* [8, 14–17] and *in vivo* [18, 19]. In two clinical trials the TZD troglitazone slowed the progression of prostate cancer within patients [16, 20], suggesting that TZDs may serve as effective therapeutic agents for prostate cancer.

Although multiple investigators have shown that TZDs suppress the growth of prostate cancer cells, the mechanism by which these compounds reduce human prostate tumor growth is not fully understood. Previous studies suggest TZD-induced decreases in prostate cancer cell proliferation are due in part to cell cycle arrest. The TZDs rosiglitazone and troglitazone increase the percentage of cells in the G_0_/G_1_ phase of the cell cycle within androgen-independent human prostate cancer cell lines [8, 14, 17]. Furthermore, exposure to the TZD troglitazone induces apoptosis in LNCaP, C4-2, and PC-3 prostate cancer cells [14, 21]. The ability of TZDs to increase apoptosis and cell cycle arrest appears to be associated with alterations in protein expression and/or activity. In PC-3 and C4-2 cells the TZDs ciglitazone, rosiglitazone, and pioglitazone increase the level of the cyclin-dependent kinase inhibitor p21 [15, 22]. TZD treatment also stimulates proteasomal degradation of cyclin D1 and *β*-catenin within human prostate cancer cells [15, 23, 24]. In addition, there is decreased phosphorylation and subsequent inactivation of retinoblastoma protein (Rb) in PC-3 cells exposed to ciglitazone [15]. Data from Shiau et al. indicate troglitazone induces apoptosis in PC-3 cells by reducing the activity of the antiapoptotic proteins Bcl-2 and Bcl_XL_ [21]. Troglitazone also reduces levels of c-Myc, a protein that plays a critical role in cell cycle progression and apoptosis, within PC-3 and C4-2 cells [14]. Therefore, TZDs modulate the level and function of several proteins that control proliferation of human prostate cancer cells.

 It is not clear whether modulation of growth factor signaling pathways contribute to TZD-induced alterations in prostate cancer cell proliferation. However, one signaling pathway that plays a critical role in the regulation of cancer growth and progression is the mitogen-activated protein kinase (MEK)/extracellular signal regulated protein kinase (Erk) signaling cascade. Upon binding ligand, growth factor receptors induce the phosphorylation of MEK which then phosphorylates the downstream kinases Erk 1 and Erk 2. The active, phosphorylated forms of Erk are then able to phosphorylate several proteins within the cytosol and nucleus to regulate cell cycle progression and apoptosis (reviewed in [25] and [26]). Gioeli et al. demonstrated that the amount of active phosphorylated Erk 1 and Erk 2 increases with increasing grade and stage of human prostate cancer [27]. Higher levels of activated Erk are also present in the more aggressive androgen-independent prostate cancer cells [28]. TZDs have been reported to regulate Erk activation in normal epithelium and cancer cells. Both troglitazone and ciglitazone increase Erk phosphorylation in the rat GN4 liver epithelial cells [29]. Troglitazone also induces Erk phosphorylation in the HCT-116 human colorectal cancer cell line [30]. Increases in Erk activation have been associated with the ability of TZDs to regulate cancer cell activity. Erk activation was associated with ciglitazone-induced increases in MMP-2 activity within HT1080 fibrosarcoma cells [31]. Ciglitazone also increases Erk phosphorylation and induces apoptosis in the HT-29 colon cancer cell line [32]. Furthermore, in NCI-H23 human nonsmall cell lung cancer cells, troglitazone-stimulated increases in apoptosis were accompanied by an elevation in Erk 1/2 phosphorylation [33]. To determine whether the anti-proliferative effects of troglitazone in prostate cancer cells are associated with altered Erk 1/2 activity, we examined whether troglitazone regulates Erk phosphorylation in the present study. Our data indicate that troglitazone does enhance Erk phosphorylation. However, this increase in Erk phosphorylation plays a minimal role in the anti-proliferative effect of troglitazone within human prostate cancer cells.

## 2. Matrials and Methods

### 2.1. Materials

Penicillin/streptomycin solution and DMEM/F12 media were purchased from Invitrogen. Dimethylsulphoxide (DMSO) was purchased from Sigma Aldrich. Fetal bovine serum was purchased from HyClone. Zap-Oglobin and Isoton II Diluent were purchased from Beckman Coulter Inc. The MEK inhibitor U0126 was purchased from Promega Corp. The PPAR*γ* antagonist GW9662 was purchased from Cayman Chemicals. Horseradish peroxidase-conjugated donkey anti-rabbit and sheep anti-mouse secondary antibodies were purchased from Amersham Biosciences. All tissue culture plasticware and additional chemicals were purchased from Fisher Scientific.

### 2.2. PPAR*γ* Agonists

The compounds troglitazone, rosiglitazone, and pioglitazone were obtained from Cayman Chemicals. To prepare stock solutions of these compounds, each drug was diluted in 100% DMSO. All stock solutions were stored at −20°C.

### 2.3. Cell Culture

The PC-3 cell line was obtained from ATCC (Rockville, MD). PC-3 cells were grown in DMEM/F-12 media supplemented with 10% FBS and 1% penicillin/streptomycin. Cell cultures were maintained in a 37°C incubator in an atmosphere supplied with 5% CO_2_.

### 2.4. Western Blot Analysis

To examine the effect of PPAR*γ* agonists on Erk phosphorylation and total Erk levels, cells were plated in 10 cm dishes at a density of 750,000 cells/dish and allowed to attach for 48 hours. The cells were next placed in 10 mL of serum free media (DMEM/F-12) for 24 hours. Cells were then treated with vehicle (100% ethanol or DMSO) or the PPAR*γ* ligands rosiglitazone, pioglitazone, or troglitazone (0–40 *μ*M) for the indicated times. In a subset of experiments, cells were pretreated for one hour with 10 *μ*M GW9662 or 10 *μ*M U0126 prior to the addition of PPAR*γ* ligand. The cells were then harvested by scraping and lysed in RIPA buffer containing 1 mM sodium vanadate and 0.6 mM phenylmethylsulfonyl fluoride (PMSF). The protein concentration of each sample was determined by using the Bradford protein assay (BioRad). Equal amounts of protein (50–100 *μ*g) from each sample were separated on SDS-PAGE gels and transferred to a nitrocellulose membrane. Membrane blots were initially blocked in TBST (1X TBS, 0.1% Tween 20) containing 5% nonfat powdered milk (total ERK 1/2) or 5% BSA (phospho-ERK 1/2). The membranes were then incubated with primary antibody overnight at 4°C. The primary antibodies used were the phospho-ERK 1/2 rabbit monoclonal antibody (Cell Signaling; 1 : 1000) and total ERK 1/2 antibody (Cell Signaling, 1 : 1000). Blots exposed to each Erk antibody were washed in TBST-5% BSA or TBST-5% milk and incubated with donkey anti-rabbit HRP secondary antibody (GE Healthcare, 1 : 5,000) diluted in TBST for one hour. The blots were then washed in TBST, incubated with ECL or ECL Plus solution according to the manufacturer's instructions (GE Healthcare) and exposed to BioMax Light autoradiography film (Kodak). Blots were stripped and reprobed with an actin antibody (Millipore) as a loading control. The UN-SCAN-IT program (Silk Scientific) was used to quantify the data from western blots.

To measure the combined effect of U0126 and GW9662 on protein expression, PC-3 cells were plated in DMEM/F-12 +10% FBS supplemented with 1% penicillin/streptomycin solution at a density of 500,000–750,000 cells/10 cm dish and allowed to attach overnight. The cells were then treated with DMSO vehicle or troglitazone (10–40 *μ*M) in the presence or absence of 10 *μ*M U0126 for 24 hours. Cell lysates were prepared and run on SDS-PAGE gels as described above. Western blot analysis was then performed as described in [14] to detect the level of c-Myc in each cell lysate. To measure p21 levels, blots were initially blocked in TBST containing 5% non-fat powdered milk and then incubated with the primary p21 antibody (Cell Signaling, 1 : 2000) overnight at 4°C. Blots were next washed in TBST-5% milk and incubated with sheep anti-mouse HRP secondary antibody (GE Healthcare, 1 : 5,000) diluted in TBST for one hour. After this incubation, each blot was washed in TBST, incubated with ECL reagent and exposed to BioMax Light autoradiography film. Blots were stripped and reprobed with an actin antibody (Millipore) to confirm equal gel loading.

### 2.5. Luciferase Reporter Assays

PC-3 cells were plated at a density of 75,000–100,000 cells per well of a six-well tissue culture plate and allowed to attach overnight. The next day, the lipofectamine reagent (Invitrogen) was used to transfect cells with CMV *β*-galactosidase reporter construct (0.05 *μ*g/well) and the PPRE-luciferase reporter plasmid (0.5 *μ*g/well). After a four-hour incubation, the lipofectamine/DNA mix was removed and the media changed to DMEM/F12 + 10% FBS. Twenty-four hours following transfection, the cells were treated with DMSO vehicle or varying concentrations of troglitazone (2.5–40 *μ*M) in the presence or absence of 10 *μ*M U0126 or 10 *μ*M GW9662 for twenty-four hours. For cells exposed to troglitazone + U0126 or troglitazone + GW9662, U0126 and G9662 were added one hour prior to the addition of troglitazone. The luciferase activity in treated cells was then measured using the Dual Luciferase Assay System kit (Promega) and normalized to the level of *β*-galactosidase activity.

### 2.6. Cell Proliferation Assays

For cell count assays, PC-3 cells were plated in six-well plates at a density of 10,000 cells per well in DMEM/F-12 media supplemented with 10% FBS and 1% penicillin/streptomycin solution. The next day, the cells were exposed for six days to DMSO vehicle or different concentrations of troglitazone (2.5–40 *μ*M) in the presence or absence of U0126 (10 *μ*M). In cells exposed to troglitazone + U0126, U0126 was added one hour prior to the addition of troglitazone. Every three days the media was changed and fresh drug was added. Following treatment, the cells were washed in Hank's balanced salt solution (HBSS) and detached from the wells using 0.25% trypsin-EDTA. The number of cells in each well was then counted using a Coulter Z1 cell counter (Beckman Coulter Inc.).

For [^3^H]-thymidine incorporation assays, PC-3 cells were plated in six-well plates at a density of 10,000 cells per well in DMEM/F12 media supplemented with 10% FBS and 1% penicillin/streptomycin solution. After allowing them to attach overnight, the cells were then exposed to DMSO or the indicated concentration of troglitazone (2.5–40 *μ*M) for 2–6 days. In experiments involving U0126, cells were exposed to 10 *μ*M U0126 for one hour prior to the addition of troglitazone. After treatment, the cells were pulsed with [^3^H]-thymidine (60–90 Ci/mmol, MP Biomedical) for 1.5 hours. The level of incorporated [^3^H]-thymidine was then measured by scintillation counter.

### 2.7. Statistical Analysis

Each experiment was performed at least three times, and representative data are shown for each experiment. One-way analysis of variance (ANOVA) was used to detect the differences between control and treated groups. ANOVAs were performed using the Sigma Stat 3.1 program (Systat Software Inc.).

## 3. Results and Discussion

### 3.1. Troglitazone Induces Erk Phosphorylation in PC-3 Cells

**Figure 1:**
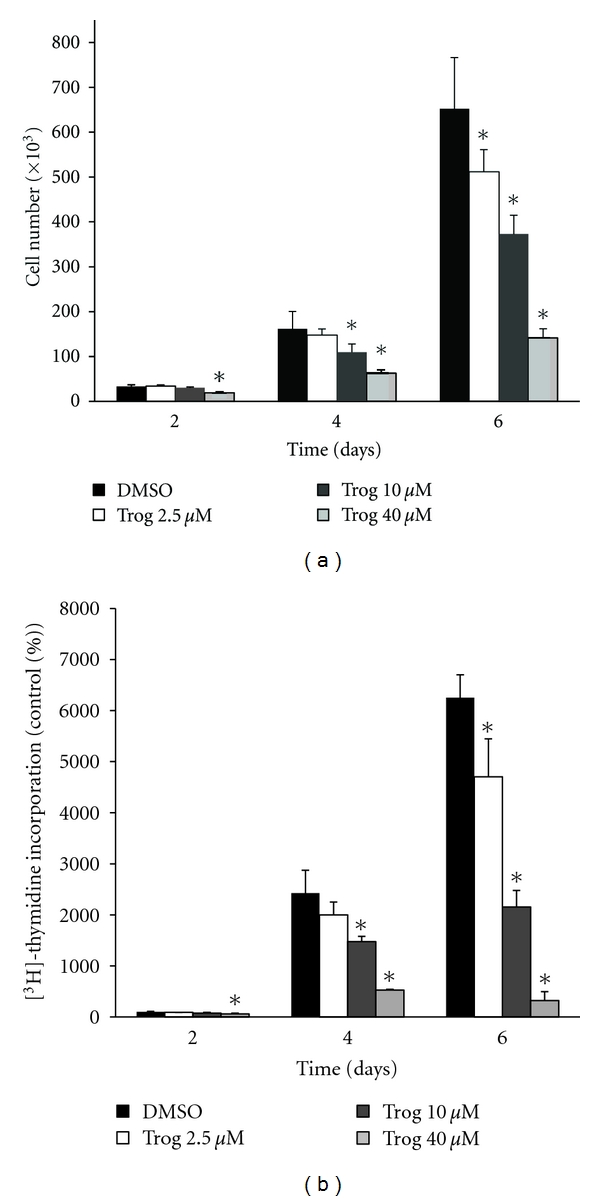
Troglitazone inhibits proliferation of PC-3 human prostate cancer cells. (a) PC-3 cells were plated in 6-well plates at 10,000 cells/well and treated for up to six days with DMSO vehicle or troglitazone (2.5–40 *μ*M). Following treatment, the cells in each well were detached using trypsin-EDTA and counted using a Coulter Counter. (b) PC-3 cells were plated in 6 well plates at 10,000 cells/well and treated for up to six days with DMSO vehicle or troglitazone (2.5–40 *μ*M). Following treatment, the cells were pulsed with [^3^H]-thymidine for 1.5 hours. The level of [^3^H]-thymidine incorporation was then measured using a scintillation counter. In both panels, each bar represents the mean ± SD for three wells. *P < 0.05 compared to DMSO vehicle at each time point. A representative experiment is shown.

**Figure 2:**
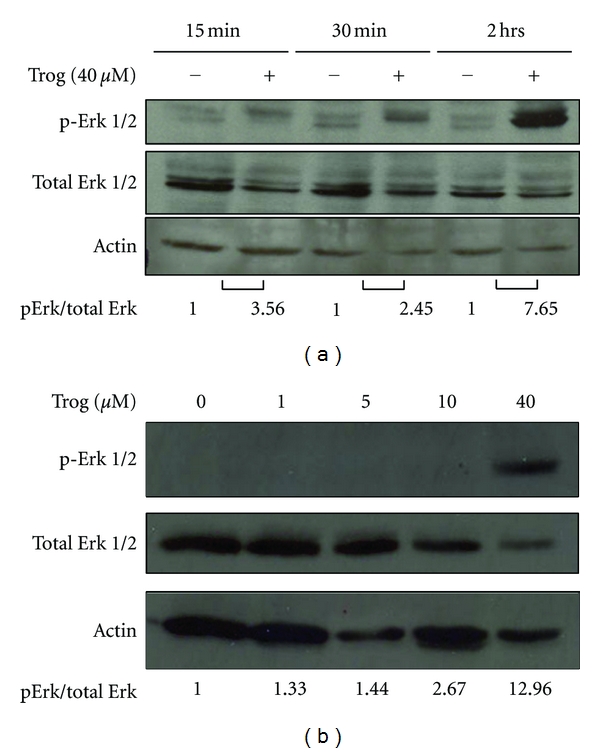
Troglitazone induces phosphorylation of ERK 1/2 MAPK in human prostate cancer cells. (a) PC-3 cells plated in serum free DMEM/F-12 media were treated with DMSO vehicle (−) or troglitazone 40 *μ*M (+) for different times (0–2 hours). The level of phosphorylated Erk 1/2, total ERK 1/2, and actin in treated cells was then measured by western blot. The data from each blot was quantified using the UN-SCAN-IT program and expressed related to the signal present in control cells for each time point. (b) PC-3 cells were treated for two hours with either DMSO vehicle or varying concentrations of troglitazone (1–40 *μ*M). Western blotting was used to measure the level of phosphorylated and total ERK 1/2 as well as actin protein in treated cells.

Our previous work has shown proliferation of C4-2 cells, an androgen-independent derivative of the LNCaP cell line, is dramatically inhibited by troglitazone [14]. Micromolar concentrations of troglitazone also significantly reduced proliferation of the androgen-independent PC-3 human prostate cancer cell line (Figure 1). Troglitazone produced a time- and dose-dependent decrease in PC-3 cell number and the level of [^3^H]-thymidine incorporation. At each time point tested, the greatest decrease in cell number and [^3^H]-thymidine incorporation was produced by a concentration of 40 *μ*M troglitazone. To determine whether concentrations of troglitazone that inhibit proliferation also regulate Erk phosphorylation, we performed a series of western blot analyses. Troglitazone at a concentration of 40 *μ*M did induce phosphorylation of Erk 1/2 in the PC-3 cell line. Elevated phosphorylation of Erk 1/2 was noted as early as fifteen minutes following treatment with troglitazone. However, this increase was more pronounced after two hours of troglitazone exposure (Figure 2(a)). The induction of Erk phosphorylation was also dose dependent. Over the concentration range tested, there was little to no increase in Erk phosphorylation at troglitazone concentrations less than 40 *μ*M. However, there was a robust induction of Erk phosphorylation in cells exposed to 40 *μ*M troglitazone (Figure 2(b)).

To determine whether this response was unique to the PC-3 cell line, we tested the effect of troglitazone on Erk activation in the C4-2 cell line. Troglitazone at a concentration of 40 *μ*M was also effective at inducing phosphorylation of Erk within C4-2 cells (data not shown). Troglitazone has been shown to induce Erk phosphorylation in breast [34], colon [30], and nonsmall cell lung cancer cells [33]. However, to our knowledge this is the first study to report that troglitazone induces Erk phosphorylation within human prostate cancer cells.

### 3.2. Troglitazone-Mediated Increases in Erk Phosphorylation Do Not Require PPAR*γ*

**Figure 3:**
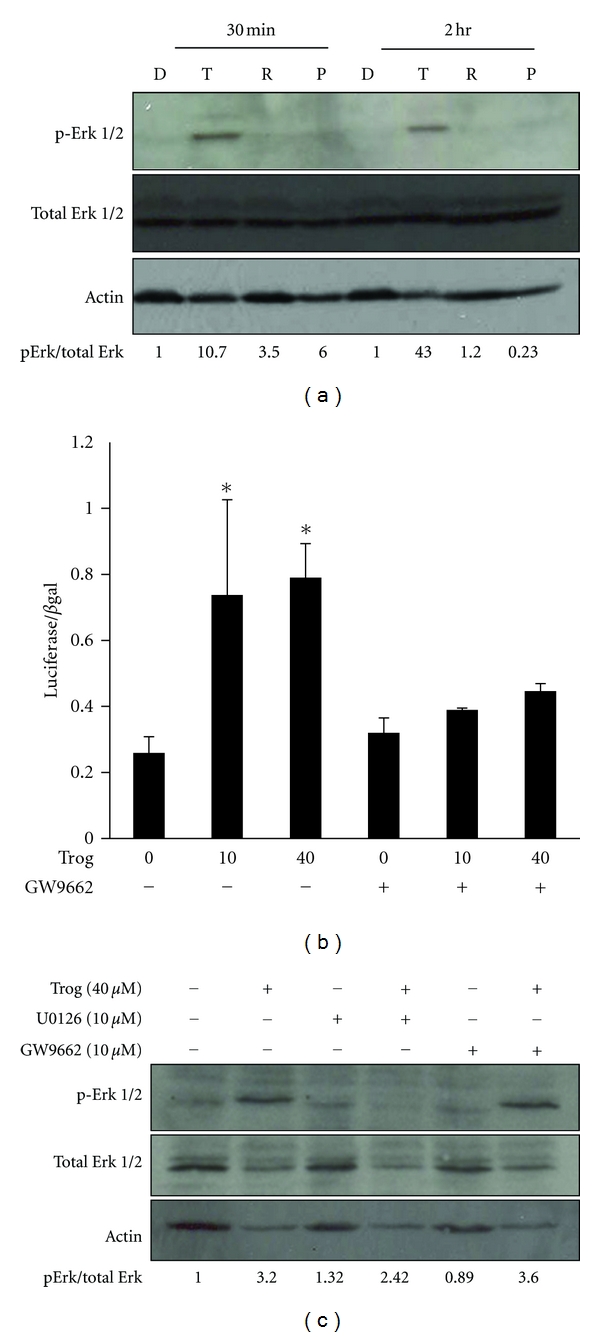
Troglitazone-stimulated increases in Erk phosphorylation occur independently of PPAR*γ*. (a) PC-3 cells were treated for 30 minutes or two hours with DMSO vehicle control (D) or the PPAR*γ* ligands troglitazone (T; 40 *μ*M), rosiglitazone (R; 40 *μ*M), or pioglitazone (P; 30 *μ*M). The level of ERK 1/2 (both total and phosphorylated) and actin was then detected by Western blot analysis. (b) PC-3 cells were first transfected with the PPRE-luciferase and CMV-*β*-galactosidase reporter plasmids. Cells were then treated for 24 hours with DMSO vehicle or troglitazone (10 or 40 *μ*M) in the presence or absence of the PPAR*γ* antagonist GW9662 (10 *μ*M). The level of luciferase activity in treated cells was measured and normalized to *β*-galactosidase activity. Each bar represents the mean SD for three wells. *P < 0.05 compared to vehicle control (0 Trog, − GW9662). (c) PC-3 cells were treated for two hours with DMSO vehicle (−) or 40 *μ*M troglitazone (+) in the presence or absence of the MEK inhibitor U0126 (10 *μ*M) or the PPAR*γ* antagonist GW9662 (10 *μ*M). Western blotting was then used to measure Erk and actin levels within treated cells. The data from each blot were quantified and expressed relative to the signal present in the vehicle control sample (−Trog, −U0126, −GW9662).

The fact that only high concentrations of troglitazone were effective at inducing Erk phosphorylation led us to suspect that this response might be due to activation of a PPAR*γ*-independent signaling pathway. To define the role of PPAR*γ* in troglitazone-induced Erk phosphorylation, we first tested whether other TZDs were equally effective at inducing activation of Erk within PC-3 cells. While troglitazone strongly induced Erk phosphorylation, we saw no increase in Erk phosphorylation in PC-3 cells exposed to comparable concentrations of the TZDs rosiglitazone or pioglitazone for two hours (Figure 3(a)). We next examined whether the PPAR*γ* antagonist GW9662 altered troglitazone-stimulated Erk phosphorylation. Luciferase assays demonstrated that GW9662 at a concentration of 10 *μ*M inhibited activation of PPAR*γ* within PC-3 cells (Figure 3(b)). However, GW9662 alone did not dramatically alter the phosphorylation state of Erk 1/2. Furthermore, this concentration of GW9662 did not prevent the increase in Erk phosphorylation produced by troglitazone (Figure 3(c)). 

Taken together, these data suggest PPAR*γ* activation is not required for troglitazone to increase Erk phosphorylation in PC-3 cells. TZDs also appear to phosphorylate Erk via a PPAR*γ*-independent pathway in breast cancer cells. Erk phosphorylation in MCF7 breast cells is increased by Δ2-TGZ, a troglitazone derivative that does not activate PPAR*γ* [34]. However, data from Li et al. demonstrated that siRNA-mediated reductions in PPAR*γ* prevent troglitazone activation of Erk in the NCI-H23 nonsmall cell lung cancer cell line [33]. Thus, while a PPAR*γ*-independent pathway mediates troglitazone-induced Erk phosphorylation in PC-3 cells, PPAR*γ* can in certain cell lines play a critical role in TZD-induced Erk phosphorylation.

### 3.3. MEK Inhibition Prevents Troglitazone-Induced Erk Phosphorylation but Does Not Affect PPAR*γ* Activation

In many cases, Erk is activated via phosphorylation by the MAPKK MEK. To determine whether MEK plays a role in troglitazone induced phosphorylation of Erk, we tested whether this response was altered in the presence of the MEK inhibitor U0126. U0126 at a concentration of 10 *μ*M did not dramatically alter the basal level of Erk phosphorylation in PC-3 cells. However, pretreatment with U0126 reduced the amount of Erk phosphorylation produced by troglitazone in the PC-3 cell line (Figure 3(c)).

**Figure 4:**
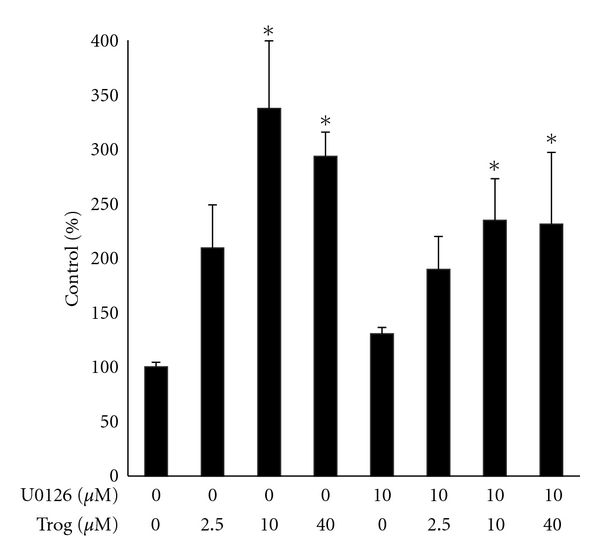
U0126 does not alter the transcriptional activity of PPAR*γ* in prostate cancer cells. PC-3 cells were plated in 6-wells plates and transfected with the PPRE-luciferase and CMV *β*-galactosidase reporter plasmids. The transfected cells were then treated for twenty-four hours with DMSO vehicle or troglitazone (2.5–40 *μ*M) in the presence or absence of 10 *μ*M U0126. Luciferase activity in treated cells was measured and normalized to *β*-galactosidase activity. Each bar represents the mean ± SD for three wells. *P < 0.05 compared to vehicle control (0 *μ*M U0126, 0 *μ*M troglitazone). At each concentration of troglitazone tested, there was no statistically significant difference noted between the troglitazone and troglitazone + U0126 groups. A representative experiment is shown.

*In vitro* Erk phosphorylates the *N*-terminus of PPAR*γ*, which consequently decreases the ability of PPAR*γ* to regulate transcription and protein expression [35–37]. To determine whether Erk phosphorylation influences the ability of troglitazone to regulate PPAR*γ* function in prostate cancer cells, we measured PPAR*γ* transcriptional activity in PC-3 cells in the presence of U0126. Compared to other human prostate cancer cell lines, PC-3 cells express a significant amount of functional PPAR*γ* protein [38]. Therefore, in these studies we transfected PC-3 cells with the PPRE-luciferase reporter to examine endogenous PPAR*γ* activity. Troglitazone produced a dose dependent increase in PPAR*γ* activation, with the greatest increase in luciferase activity occurring at troglitazone concentrations >10 *μ*M (Figure 4). U0126 alone produced a slight increase in basal PPAR*γ* transcriptional activity that was not statistically significant. In addition, the ability of troglitazone to increase PPRE-luciferase activity was not altered in cells pretreated with U0126. Therefore, troglitazone-induced increases in Erk phosphorylation within PC-3 cells do not appear to reduce this TZD's ability to activate PPAR*γ*.

### 3.4. U0126 Does Not Inhibit the Antiproliferative Effects of Troglitazone in PC-3 Cells

**Figure 5:**
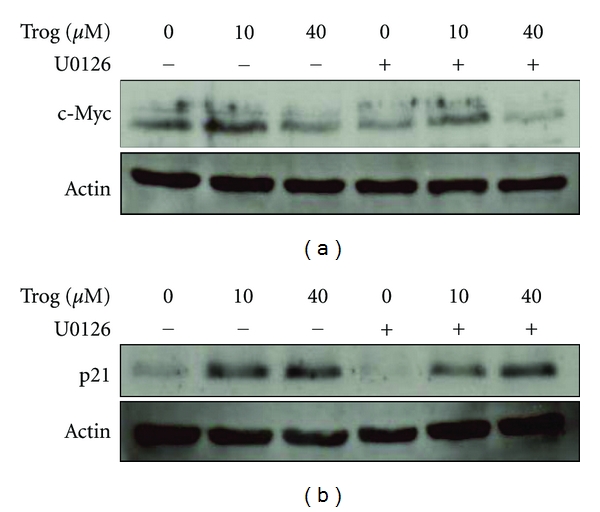
Combined effect of U0126 and troglitazone on cell cycle protein levels in PC-3 cells. PC-3 cells were plated in DMEM/F-12 media supplemented with 10% FBS and 1% penicillin/streptomycin solution and allowed to attach. The cells were then treated for twenty-four hours with DMSO vehicle or different concentrations of troglitazone in the presence (+) or absence (−) of 10 *μ*M U0126. Western blot analysis was used to measure c-Myc, p21, and actin levels within treated cells.

The anti-proliferative effects of troglitazone within human prostate cancer cells have been linked to alterations in cell cycle progression and apoptosis. Therefore, we explored whether inhibition of MEK modified the ability of troglitazone to regulate proteins that control these two processes. In these studies we focused on two proteins: p21 and c-Myc. The cyclin-dependent kinase inhibitor p21 plays a critical role in the G1/S cell cycle transition [39, 40]. Recent work form our laboratory has also shown troglitazone suppresses expression of the proto-oncogene c-Myc [14]. In PC-3 cells, treatment with troglitazone for 24 hours resulted in a significant increase in p21 protein levels. However, this induction was not altered in cells cotreated with troglitazone and U0126 (Figure 5). In a similar manner, U0126 did not interfere with the ability of troglitazone to suppress c-Myc.

**Figure 6:**
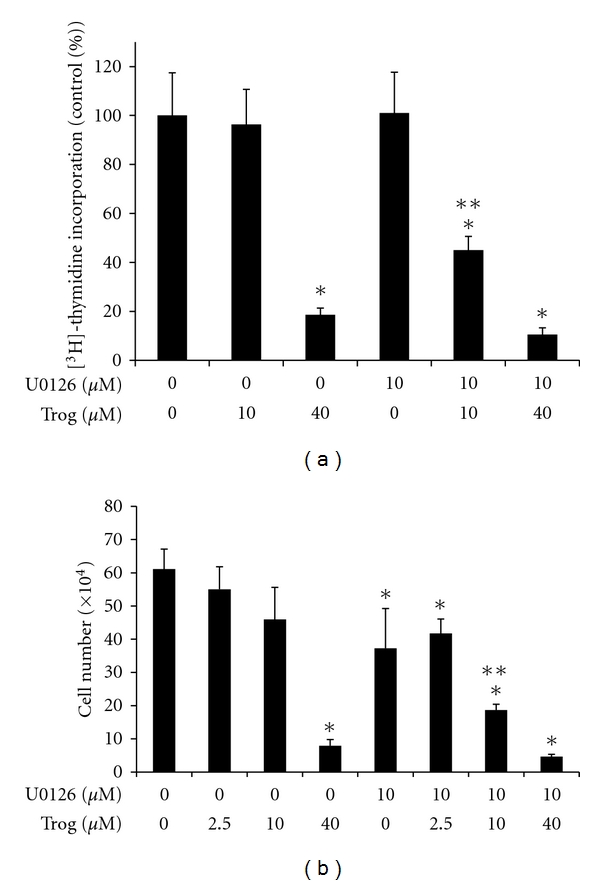
Combined effect of U0126 and troglitazone on PC-3 cell proliferation. (a) PC-3 cells were first plated in 6-well plates at a density of 10,000 cells/well. The cells were then treated with DMSO vehicle or troglitazone (10–40 *μ*M) in the presence or absence of 10 *μ*M U0126. [^3^H]-thymidine incorporation assays were then used to measure proliferation of treated cells. (b) PC-3 cells were plated in 6-well plates at a density of 10,000 cells/well and treated for six days with DMSO vehicle or troglitazone in the presence or absence of 10 *μ*M U0126. Following treatment, the cells were detached using trypsin-EDTA and counted using a Coulter Counter. For both panels, each bar represents the mean ± SD for three wells. *P < 0.05 compared to DMSO control. **P < 0.05 compared to U0126 alone and 10 *μ*M troglitazone alone.

We next tested whether U0126 alters the anti-proliferative effect of troglitazone. Cell count assays revealed treatment with either troglitazone or U0126 alone for six days reduced proliferation of the PC-3 cell line. Cotreatment with U0126 did not block the decrease in cell number produced by either 10 *μ*M or 40 *μ*M troglitazone. In fact, the combination treatment of U0126 and 10 *μ*M troglitazone decreased cell number to a greater extent than that seen with either compound alone (Figure 6(b)). We saw a similar pattern in assays where cell proliferation was measured by [^3^H]-thymidine incorporation assays. In these studies U0126 did not block the reduction in thymidine incorporation produced by micromolar concentrations of troglitazone (Figure 6(a)). These data would suggest that Erk phosphorylation does not contribute to the ability of troglitazone to suppress prostate cancer cell proliferation. At present, we do not know the reasons that underlie the greater anti-proliferative response detected in cells cotreated with U0126 and 10 *μ*M troglitazone. We and others have shown that troglitazone and other TZDs can induce apoptosis in human prostate cancer cells [14, 21]. Our preliminary studies suggest that U0126 does not enhance the ability of troglitazone to induce apoptosis within PC-3 cells. However, additional experiments are required in order to confirm this finding and to explore whether alterations in cell cycle progression contribute to this enhanced response.

Taken together, our data indicate that increases in Erk phosphorylation are not required for the growth inhibitory effects of troglitazone. This is likely due to the fact that U0126 does not prevent troglitazone's ability to modulate expression of cell cycle proteins such as p21 and c-Myc. While our studies suggest that Erk phosphorylation is not required for troglitazone-mediated changes in protein expression, this may vary depending on the cell line and PPAR*γ* ligand tested. Troglitazone increases expression of nucleobindin 2 (NUCB2) via activation of Erk in HTB185 brain medulloblastoma cells [41]. Also, Papineni et al. demonstrated blocking Erk via the MEK inhibitor PD98059 reduced the ability of the non-TZD PPAR*γ* ligand *β*-CDODA-Me to increase p21 in LNCaP prostate cancer cells [42]. Therefore, in some situations Erk does play a role in the regulation of protein expression by TZDs and other PPAR*γ* ligands.

In this study we have primarily examined MEK as an upstream regulator of Erk activity. However, MEK can regulate PPAR*γ* independently of Erk. Work by Burgermeister et al. revealed MEK physically associates with PPAR*γ* and promotes nuclear export of PPAR*γ* in a manner that does not require Erk phosphorylation [43]. Both the Erk-dependent and Erk-independent functions of MEK can be blocked by the MEK inhibitor U0126. Our experiments involving U0126 suggest that MEK is critical for troglitazone-induced phosphorylation of Erk within human prostate cancer cells. However, it is unlikely that Erk-independent functions of MEK influence the ability of troglitazone to regulate expression of cell cycle proteins and cell proliferation. Additional studies are required to confirm that MEK plays a minimal role in troglitazone-mediated responses within prostate cancer cells.

Troglitazone has been shown to not only phosphorylate Erk but also activate the MAP kinases p38 and JNK within cancer cells. Inhibition of JNK suppresses troglitazone-induced apoptosis in human breast carcinoma and hepatoma cells [44, 45]. In MCF-7 breast cancer cells p38 inhibitors also enhance the ability of troglitazone to stimulate apoptosis [45]. To date, we have not been able to detect an alteration in p38 phosphorylation in PC-3 cells following troglitazone exposure. Furthermore, preliminary data from our laboratory indicate compounds that inhibit p38 and JNK activity can alone inhibit PC-3 proliferation, but do not alter the anti-proliferative effect of troglitazone (data not shown). We therefore believe that the anti-proliferative effect of troglitazone within this prostate cancer cell line does not involve alterations in p38 and/or JNK activity.

## 4. Conclusions

In summary, our data indicate that troglitazone induce Erk phosphorylation in human prostate cancer cells via a PPAR*γ*-independent signaling pathway. Inhibition of the MEK/Erk signaling pathway prevents this phosphorylation of Erk, but does not interfere with the anti-proliferative effects of troglitazone. Of the TZDs that have been commercially available, troglitazone is the compound which has produced the most promising results in clinical studies of prostate cancer. However, concerns regarding liver toxicity have resulted in troglitazone being removed from the US market in 2000. Combination treatments which could enhance the anti-tumor effects of troglitazone while minimizing its toxicity could potentially be one way to reduce the death and suffering associated with prostate cancer. Our data demonstrate the combination of troglitazone with inhibitors of the MEK/Erk pathway does suppress proliferation of human prostate cancer cells. However, at most concentrations the response of the combination is only slightly greater than that seen with troglitazone alone. Additional studies must be performed to determine whether the anti-tumor effects of troglitazone can be further enhanced by other kinase inhibitors.
